# Use of the K factor from the Universal Soil Loss Equation can show arable land in Palau

**DOI:** 10.12688/f1000research.22229.1

**Published:** 2020-02-07

**Authors:** Masato Oda, Yin Yin Nwe, Hide Omae

**Affiliations:** 1 Japan International Research Center for Agricultural Sciences, Tsukuba, Ibaraki, 305-8686, Japan; 2 Palau Community College, Koror, Palau

**Keywords:** Babeldaob, hillside farming, island, tillage, mulching, USLE equation

## Abstract

Palau is an island in the Micronesia region of the western Pacific Ocean. The island receives heavy rainfall and has steep slopes, so 92% of the land is categorized within the most erodible rank, with a T factor of 5. A recent study reported that the water infiltration rate is proportional to the root mass of the crop soil; therefore, we attempted to evaluate the performance of root mass for preventing soil erosion. We covered parts of the land, with a slope of 15.4° (13.4°–17.3°), with weed control fabric to prevent the growth of grass and roots, then removed the fabric, cultivated the land, planted sweet potatoes, and compared the amount of soil erosion with other areas. Surprisingly, there was no erosion at all in the test plots, although there were 24 rainfall events that caused erosion. For the parameters of the Universal Soil Loss Equation (USLE) equation used in the present study, only the K factor was not actually measured. This means the K factor was larger than the actual value. Land at low risk of soil erosion and suitable for agriculture can be found by measuring K factor locally, even if the area is categorized as unsuitable.

## Introduction

Palau forms part of the Micronesia region in the western Pacific Ocean. There are open agricultural fields that were once utilized by the Japanese, prior to World War Two. The redevelopment of these fields is starting to occur. Generally, fields with inclines of more than 8° are unsuitable for growing crops, but most of the agricultural fields in Palau have slopes of more than 8°. As well as having steep slopes, the island is also subject to heavy rainfall, therefore 92% of the land is categorized within the most erodible rank, having a T factor of 5 (more than 5 tons per acre per year) ( Smith, 1983). In recent studies, the estimated risk of soil erosion from agricultural land was reported to be from 720 to 813 tons per ha per year ( USDA Natural Resources Conservation Service, 2003). No-tillage farming is effective for preventing soil erosion ( Zuazo & Pleguezuelo 2009), but the use of herbicides is unfavorable in Palau from an ecological perspective. Therefore, either tillage or the use of weed control fabric is necessary. The problem of tillage is the early stage of the crop of small vegetation coverage ( Wischmeier & Smith 1978). It is essential to increase the water infiltration rate at this stage. The water infiltration rate is positively proportional to the root mass of the crop soil ( Oda *et al.*, 2019). Here, we clarified the risk of erosion in a field with an incline typical for Palau. In addition, we clarified the aftereffects of using weed control fabric, because the use of these fabrics can reduce root mass in the tropics and may result in erosion ( Oda *et al.*, 2019).

## Methods

### Site description

The experiment was conducted at the Palau Community College Research and Development Station (N7.53, E134.56). The soil here comprises “Ngardmau-Bablethuap Complex”, which is characterized as a very gravelly loam with low organic matter content of between 1% and 4% ( Smith, 1983). The T factor is more than 5 tons per acre per year, although the permeability is moderately rapid (15–50 cm/hr) and very well drained. The available water capacity is between 0.05 and 0.10 cm/cm ( Smith, 1983). The previous crop grown on the land was taro ( *Colocasia esculenta*). The slope is 15.4° (13.4°–17.3°).

### Treatments

**Figure 1.**
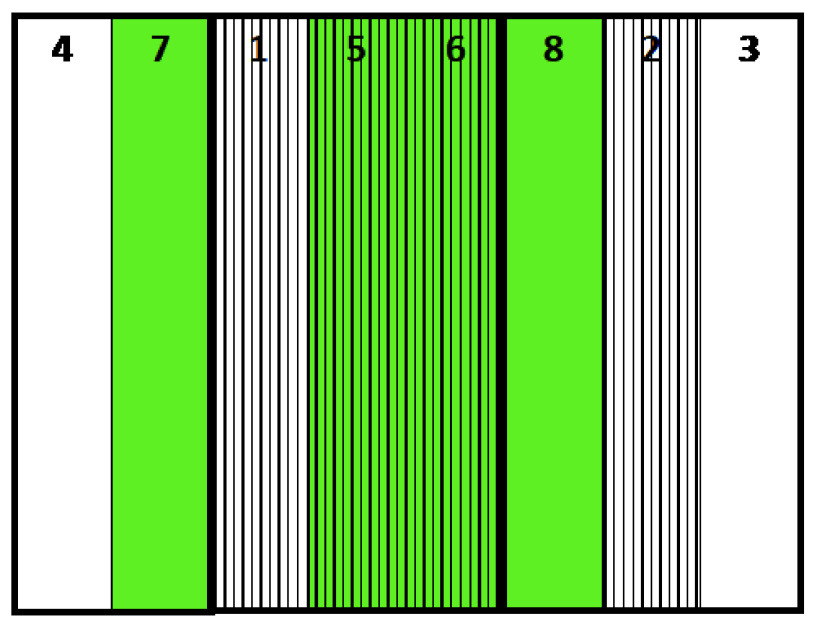
Location of plots. Green: No mulch treatment, Stripe: Ridge treatment.

**Figure 2.**
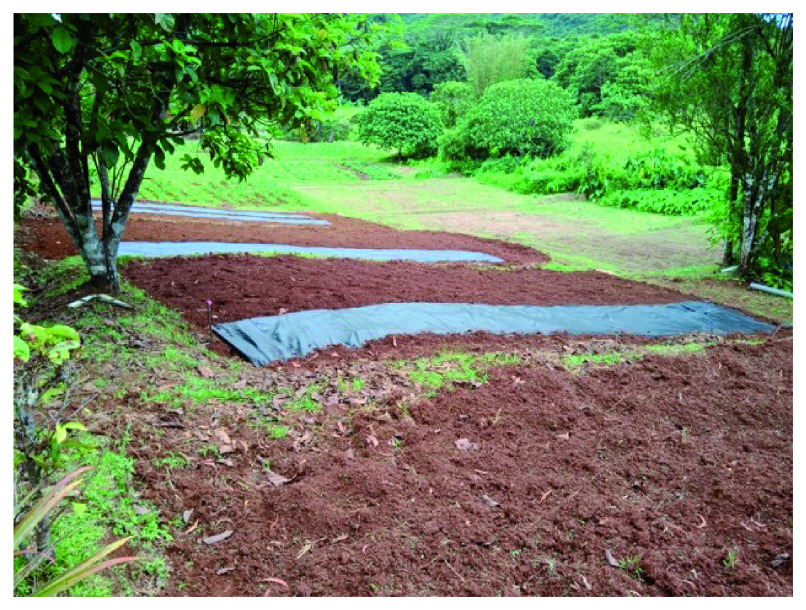
Initial condition of the field.

**Figure 3.**
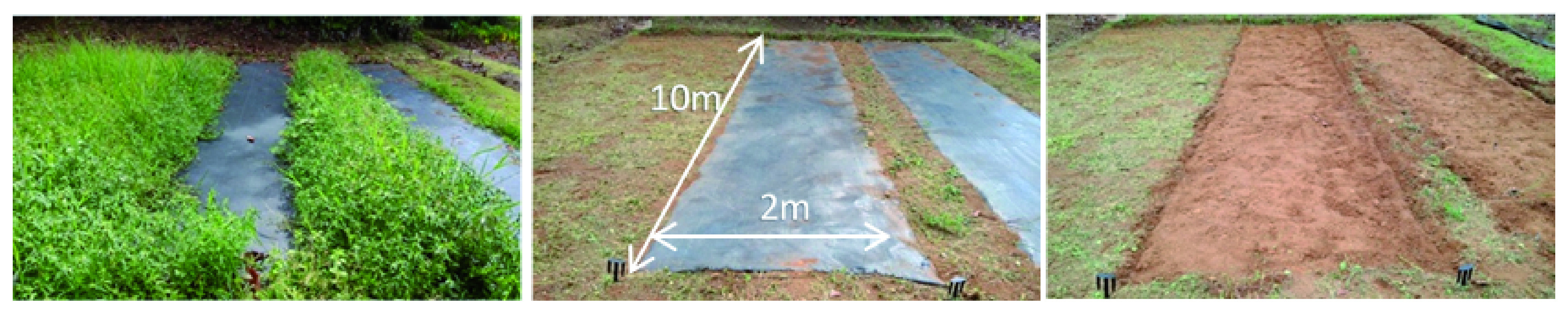
Conditions before cultivation.

**Figure 4.**
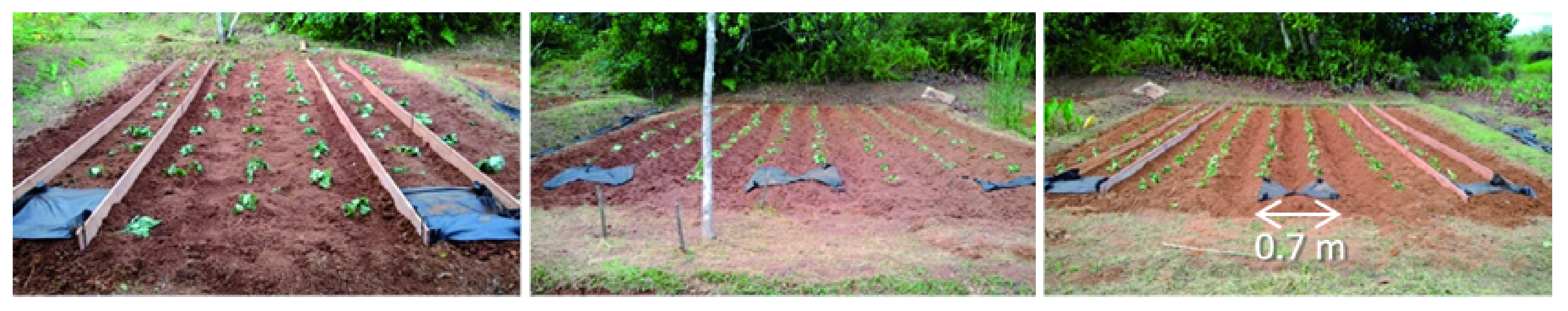
Initial conditions. The order of the plots is 4, 7, 1, 5, 6, 8, 2, 3.

We conducted the experiment from January to July 2019. The treatments were plants (with or without) × ridge (with or without) × 2 replications. We set these eight plots (2 × 10 m) randomly on the field ( Table 1, Figure 1 and Figure 2). We tilled the field using a hand tractor on 22 January, leveled the field, and covered half the plots with weed control fabric (polypropylene, 0.4-mm thick, 120 g m ^–2^; I-Agri Corp., Tsuchiura) on 28 January. We cut weeds on 16 April, blew off the residue, removed the weed control fabric on 17 April ( Figure 3), then tilled each plot using the hand tractor up and down so that the soil did not mix. The average thickness of the soil tilled was 16 cm. We made a 70 cm width of the monitoring areas in the center of the plots by ridges or wooden boards (for the no-ridge treatment). We transplanted sweet potatoes ( *Ipomoea batatas*) at 70 cm intervals on 17 April ( Figure 4). We dug trenches at the upper end of the fields to prevent rainwater inflow. We embanked the lower ends and added 1-m lengths of weed control fabric to trap any eroded soil. Fertilizer was not applied. Hand weeding was conducted on 21 May and 6 June.

**Table 1.** Treatments.

Block	Plot ID	Plants	Ridge	Slope/°
Left	4			15.1
	7	+		17.3
Mid	1		+	14.2
	5	+	+	14.6
	6	+	+	15.7
Right	8	+		13.4
	2		+	16.1
	3			16.6

### Determination

Following every heavy rainfall event, we collected any soil that had been eroded. We collected precipitation data every 5 minutes via a weather station in the Palau Community College Research and Development Station. The condition of the fields was recorded using an automatic camera.

### Analysis

We identified rainfall events that caused severe erosion (more than 3 mm/10 min) ( Onaga, 1969) and compared the amount of eroded soil of each events.

We estimated erosion using the Universal Soil Loss Equation (USLE) equation ( Wischmeier & Smith 1978).

A = R×K×LS×P×C metric ton ha ^–1^ year ^–1^

E=210+89 log _10_ I _30_ 100 metric ton ha ^–1^

I _30_ cm h ^–1^: maximum rainfall in 30 min multiplied to 60 min; rainfall less than 1.27 cm is omitted, and the maximum value is 7.62 cm.

A = EI×K×LS×P×C metric ton ha ^–1^

K = 0.15

LS = (10/20.0)^0.5 × (68.19 sin ^2^ 15.4° + 4.75sin 15.4°+0.068)= 4.34

P = 1.00; vertical ridge

C =1.0; Tillage

EI = (E×I _30_)/100

Plot area = 7 m ^2^

## Results

### Precipitation

**Figure 5.**
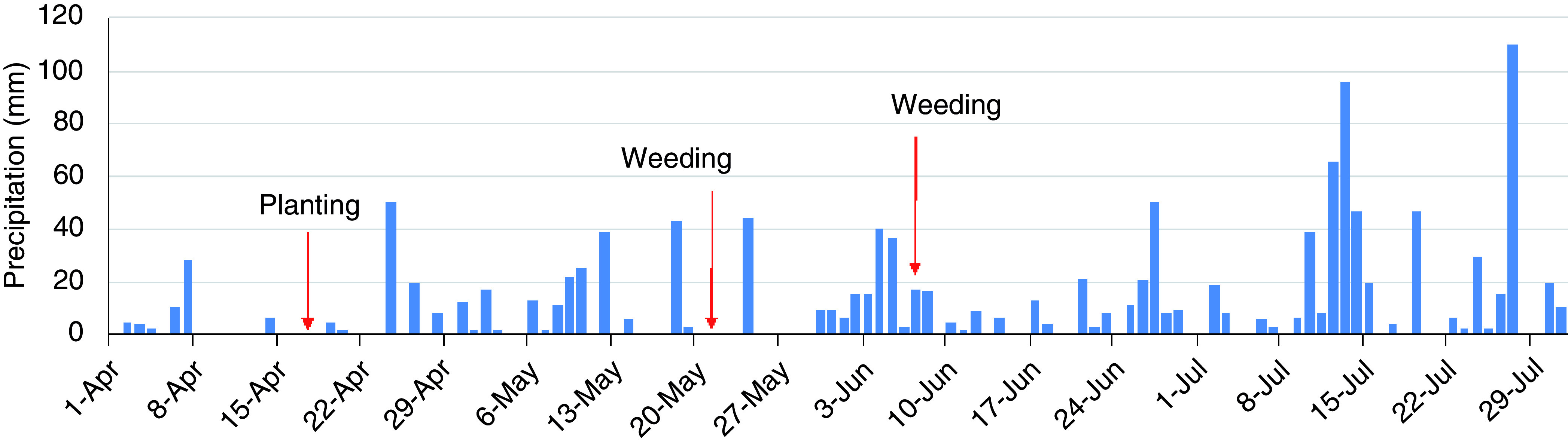
Daily precipitation.

**Figure 6.**
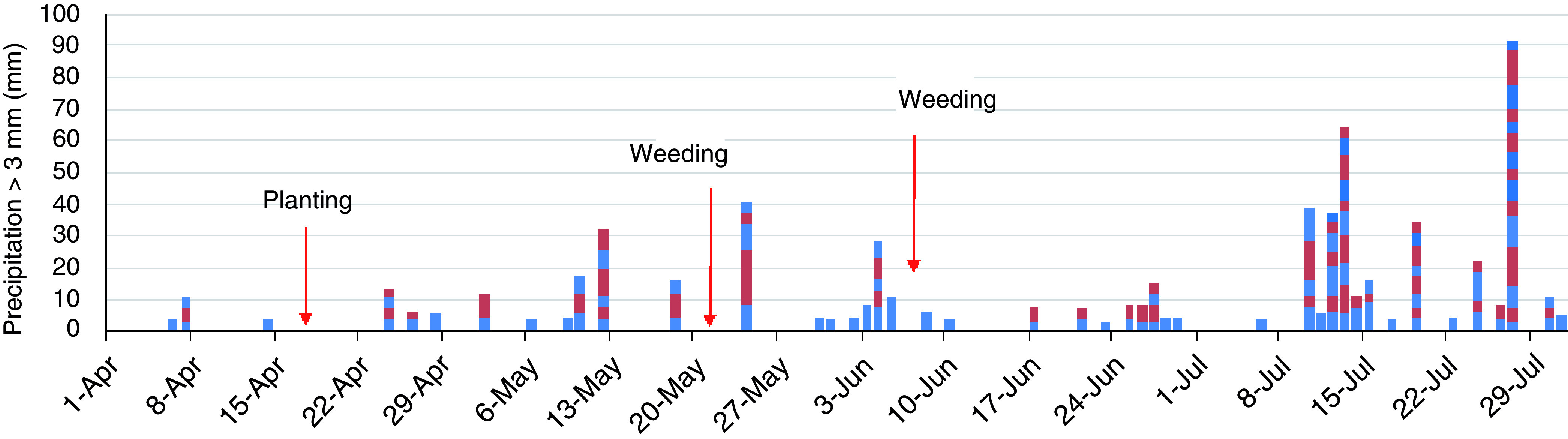
Erosive rainfall events. The blocks show a rainfall event of more than 3 mm/10 min and the amount of precipitation. The colors distinguish the events.

The field site received regular rainfall, with total precipitation of 992 mm during the experimental period, from 17 April to 15 July ( Figure 5). There were 46 days of erosive rainfall more than 3 mm per 10 min ( Figure 6). The rainfall threshold where surface runoff occurs is 2–3 mm per 10 min on a 15° slope, although these values vary according to different soil characteristics ( Onaga, 1969). There was a highly erosive rainfall event on day 7 after planting (2 May). Following weeding, an erosive period, a heavy rainfall event of 17 mm per 10 min occurred on the next day after weeding took place. The second weeding was conducted after seven days of intensive rainfall, with a further erosive rainfall event of 7 mm per 10 min that occurred just after weeding took place. Thus, the rainfall conditions during the experimental period were expected to result in severe soil erosion.

### Estimated erosion

There were 24 rainfall events that could have caused erosion during the observation period ( Table 2). The estimated erosion was 0.57 kg of erosion per plot on day 7, the first rainfall event, after transplanting. That was 2.82 kg after first weeding.

**Table 2.** Estimated erosion following each rainfall event.

Date	Day	I _30_ cm h ^–1^	E	EI	A t/ha	Erosion kg/plot	Remark
8-Apr	–9	1.76	232	4.08	0.89	0.62	(Before planting)
**24-Apr**	**7**	**1.64**	**229**	**3.76**	**0.82**	**0.57**	**1st rain**
26-Apr	9	1.36	222	3.02	0.65	0.46	
1-May	14	2.76	249	6.88	1.49	1.05	
2-May	15	2.84	250	7.11	1.54	1.08	
6-May	19	1.52	226	3.44	0.75	0.52	
9-May	22	1.68	230	3.86	0.84	0.59	
10-May	23	2.92	251	7.34	1.59	1.12	
12-May	25	3.52	259	9.10	1.98	1.38	
18-May	31	2.92	251	7.34	1.59	1.12	
**24-May**	**37**	**6.56**	**283**	**18.55**	**4.02**	**2.82**	**After weeding**
2-Jun	46	1.56	227	3.54	0.77	0.54	
3-Jun	47	2.68	248	6.65	1.44	1.01	
5-Jun	49	2.68	248	6.65	1.44	1.01	
8-Jun	52	0.76	199	1.52	0.33	0.23	After weeding
8-Jun	52	2	237	4.74	1.03	0.72	
17-Jun	61	1.72	231	3.97	0.86	0.60	
21-Jun	65	1.12	214	2.40	0.52	0.36	
27-Jun	71	1.96	236	4.63	1.00	0.70	
29-Jun	73	1.6	228	3.65	0.79	0.55	
2-Jul	76	1.04	212	2.20	0.48	0.33	
10-Jul	84	5.36	275	14.73	3.20	2.24	
13-Jul	87	2.44	244	5.97	1.29	0.91	
14-Jul	88	4.32	267	11.52	2.50	1.75	
15-Jul	89	2.44	244	5.97	1.29	0.91	(End of observation)
19-Jul	93	1.96	236	4.63	1.00	0.70	
19-Jul	93	2.36	243	5.74	1.25	0.87	
25-Jul	99	3.04	253	7.69	1.67	1.17	
26-Jul	100	2	237	4.74	1.03	0.72	
27-Jul	101	5.4	275	14.86	3.22	2.26	
28-Jul	102	4.28	266	11.39	2.47	1.73	
30-Jul	104	1.4	223	3.12	0.68	0.47	
Sum of observation period (Day 7 to 89)					32.23	22.56	

### Soil erosion

**Figure 7.**
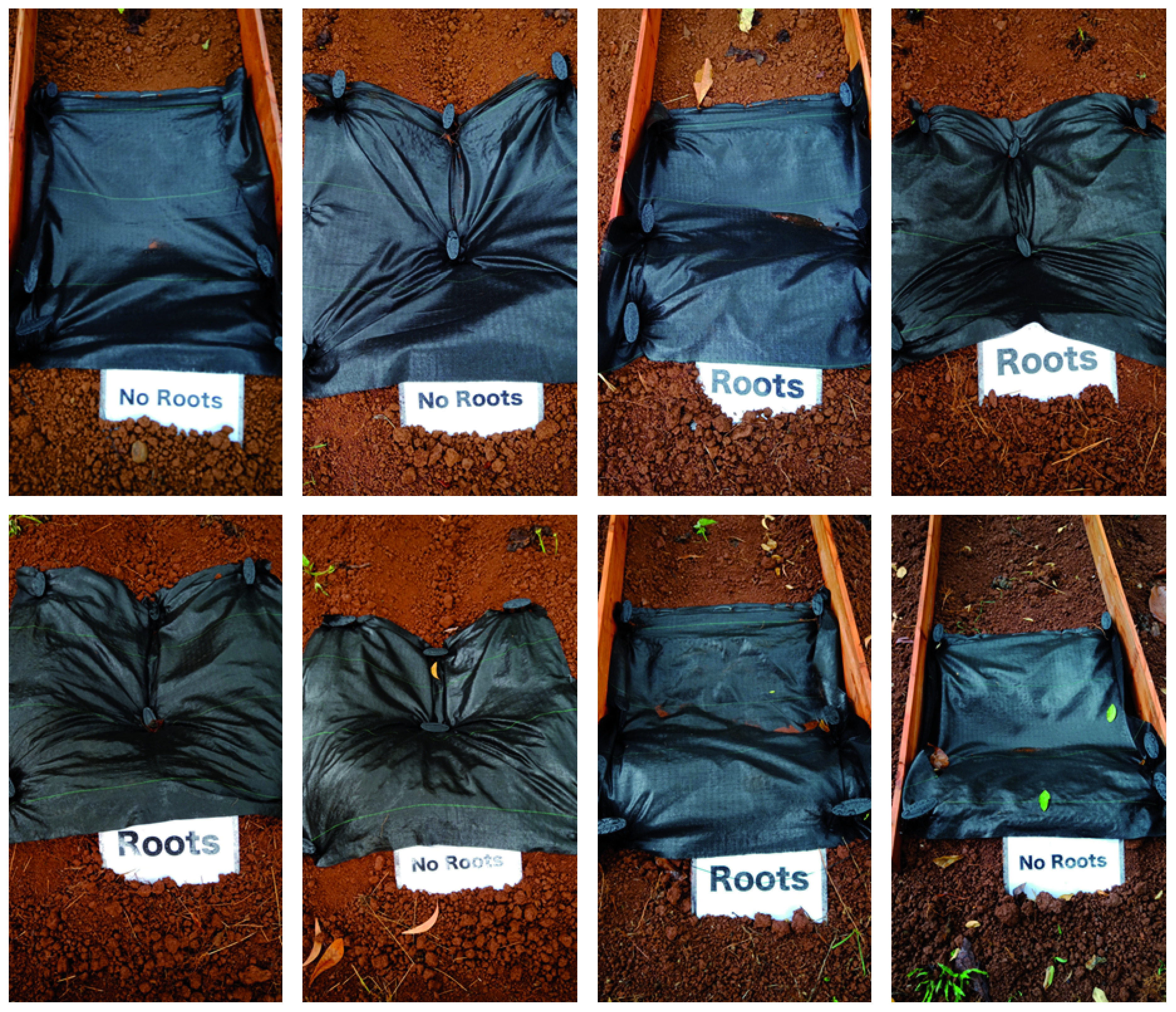
Actual erosion of the first rainfall event after transplanting. The estimated erosion was 0.57 kg per plot.

Despite the severe rainfall conditions, none of the plots had any erosion at all through the experimental period ( Figure 7).

### Vegetation coverage

**Figure 8.**
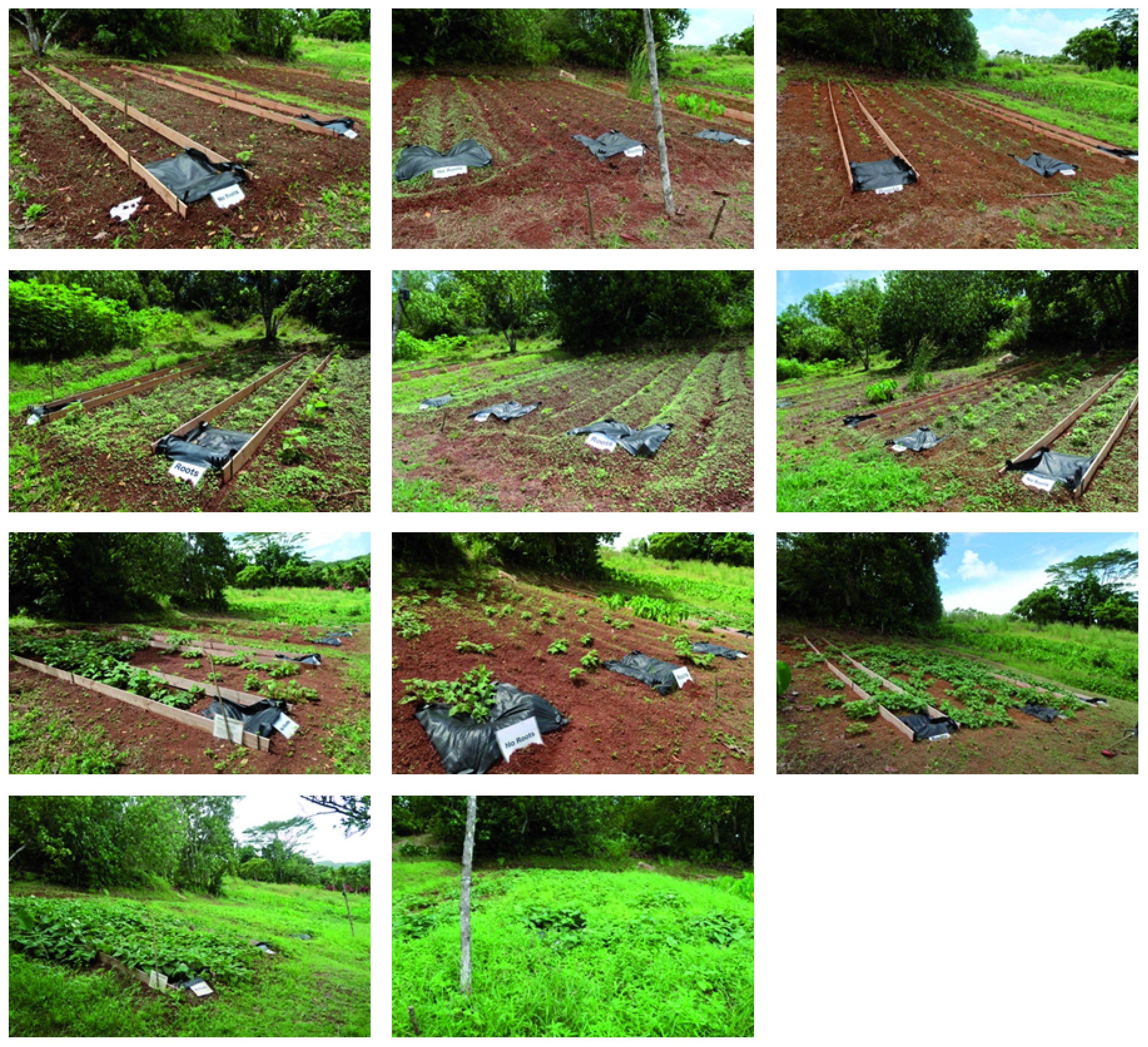
Vegetation coverage. Top panel: day 14, Upper middle panel: day 21 (before the first weeding), Lower middle panel: day 54 (after second weeding), Bottom panel day 89.

Most of the soil surface was bare by day 14 (1 May). The surface of the soil was covered by small weeds on day 21 (8 May), the day of the first weeding. The vegetation coverage by visual inspection ranged from 15–85% on day 54 (10 Jun), after the second weeding. The vegetation coverage was 100% by day 89 (15 July) ( Figure 8).

## Discussion and conclusion

The experiment was conducted under severe conditions, with a slope of approximately 15° and vertical ridge. The treatment with weed control fabric was expected to erase the effect of root mass for preventing soil erosion. There were many intensive rainfall events during the experimental period. Nevertheless, no soil erosion occurred. This means that the risk of soil erosion was low for the experimental soil in fields with slopes of less than 15°. Tillage is available. Although the use of mulching material may erase the effect of root mass for preventing soil erosion, still the use of mulching material is available.

The results were unexpected. The vertical ridge may affect the result because a vertical ridge without a catch canal is less erosive ( Shima *et al.*, 1991).

For the parameters of the USLE equation in the present study, only the K factor was not actually measured. This means that the K factor was larger than the actual value. Low erosion land for agriculture can be found by measuring erosion locally, even if the area is categorized as being unsuitable for field crops. The risk of erosion should be clarified for other soil types, and the effect of the previous crop type too. For taro, the previous crop in these fields, the roots might be left in the soil; although, we took a fallow period. Land suitable for agriculture and at low risk of soil erosion can be found in Palau by determining site-specific K factor measurements.

## Data availability

### Underlying data

Figshare: Precipitation of Palau, https://doi.org/10.6084/m9.figshare.11769909.v1 ( Oda *et al.*, 2020). Data are available under the terms of the Creative Commons Zero "No rights reserved" data waiver (CC0 1.0 Public domain dedication).
