# Locally measured USLE K factor expands sustainable agricultural land in Palau

**DOI:** 10.12688/f1000research.22229.3

**Published:** 2021-06-22

**Authors:** Masato Oda, Yin Yin Nwe, Hide Omae

**Affiliations:** 1 Japan International Research Center for Agricultural Sciences, Tsukuba, Ibaraki, 305-8686, Japan; 2 Palau Community College, Koror, Palau

**Keywords:** Babeldaob, hillside farming, island, tillage, mulching, USLE equation

## Abstract

From the viewpoint of sustainability, annual soil erosion should be controlled below an erosion level. Palau is an island in the Micronesia region of the western Pacific Ocean. The island receives heavy rainfall and has steep slopes, so 80% of the land is categorized within the most fragile rank (T factor = 1) in soil erosion. We tested several methods of preventing soil erosion on the land, with a slope of 15.4° (13.4°–17.3°), cultivated the land, planted sweet potatoes, and compared the amount of soil erosion. Surprisingly, there was no erosion at all in all plots (including control plots), although 24 rainfall events occurred and the USLE equation predicted 32 tons per ha of soil erosion in the cropping period. For the parameters of the USLE equation used in this study, only the K factor was not measured (cited from a USDA report). Namely, the K factor estimated by soil texture was larger than the actual value. Measuring the K factor in the fields can expand Palau's sustainable agricultural land.

## Introduction

From the viewpoint of sustainability, annual soil erosion should be controlled below an erosion level of the T factor ( USDA Natural Resources Conservation Service). Although No-tillage farming is effective for preventing soil erosion ( Zuazo & Pleguezuelo, 2009) but the use of herbicides is unfavorable from an ecological perspective; therefore, reducing soil erosion in tillage farming is needed. The erosion caused by tillage occurs with small vegetation coverage in the early stage of the crop ( Wischmeier & Smith, 1978). It is essential to increase the water infiltration rate at this stage. The water infiltration rate is positively proportional to the root mass of the crop soil ( Oda *et al.*, 2019). Therefore, we tried clarifying the risk of erosion and the effect of root mass for preventing soil erosion in a field with an incline typical for Palau in an area categorized as highly erodible. Surprisingly, there was no erosion at all in all plots. The results show that land at low risk of soil erosion can be found by determining site-specific K factor measurements. Although we failed to evaluate the effects of the treatments, this information is important for Palau’s agricultural development.

## Methods

### Site description

Palau forms part of the Micronesia region in the western Pacific Ocean. Palau's economy is mainly due to tourism and the increase in tourists increases the consumption of agricultural products. Palau imports them, but it is preferable to produce them domestically. The agriculture in Palau is mainly taro cultivation at swamp by a traditional and environmentally friendly method. Before World War II, Japanese settlers developed agricultural land. In recent years, the redevelopment of these fields using modern farming methods has begun. Fields with inclines of more than 8° are unsuitable for growing crops, but most of the agricultural fields in Palau have slopes of more than 8°. The island is also subject to heavy rainfall (ca. 3300 mm – 3900 mm). USDA Natural Resources Conservation Service in 2009 categorized most of the land (80%) as the most fragile rank (T factor = 1). T Factor values range from 1 ton/acre/year for the most fragile soils, to 5 tons/acre/year for soils that can sustain more erosion without losing significant productive potential ( USDA Natural Resources Conservation Service). A study estimated the risk of soil erosion from agricultural land was reported to be from 720 to 813 tons per ha per year ( Gavenda *et al.*, 2005).

The experiment was conducted at the Palau Community College Research and Development Station ( N7.529694, E134.560522). The station is located in the interior of Babeldaob island, the second largest of the Micronesian islands, and is surrounded by forest. The field is one of the agricultural fields that were once used by the Japanese settlers. The soil here is Oxisol (ferralsols)—“Ngardmau-Bablethuap Complex,” which is characterized as a very gravelly loam with low organic matter content of between 1% and 4%. The permeability is moderately rapid (15–50 cm/hr) and very well drained. The available water capacity is between 0.05 and 0.10 cm/cm ( Smith, 1983).

### Treatments

**Figure 1.**
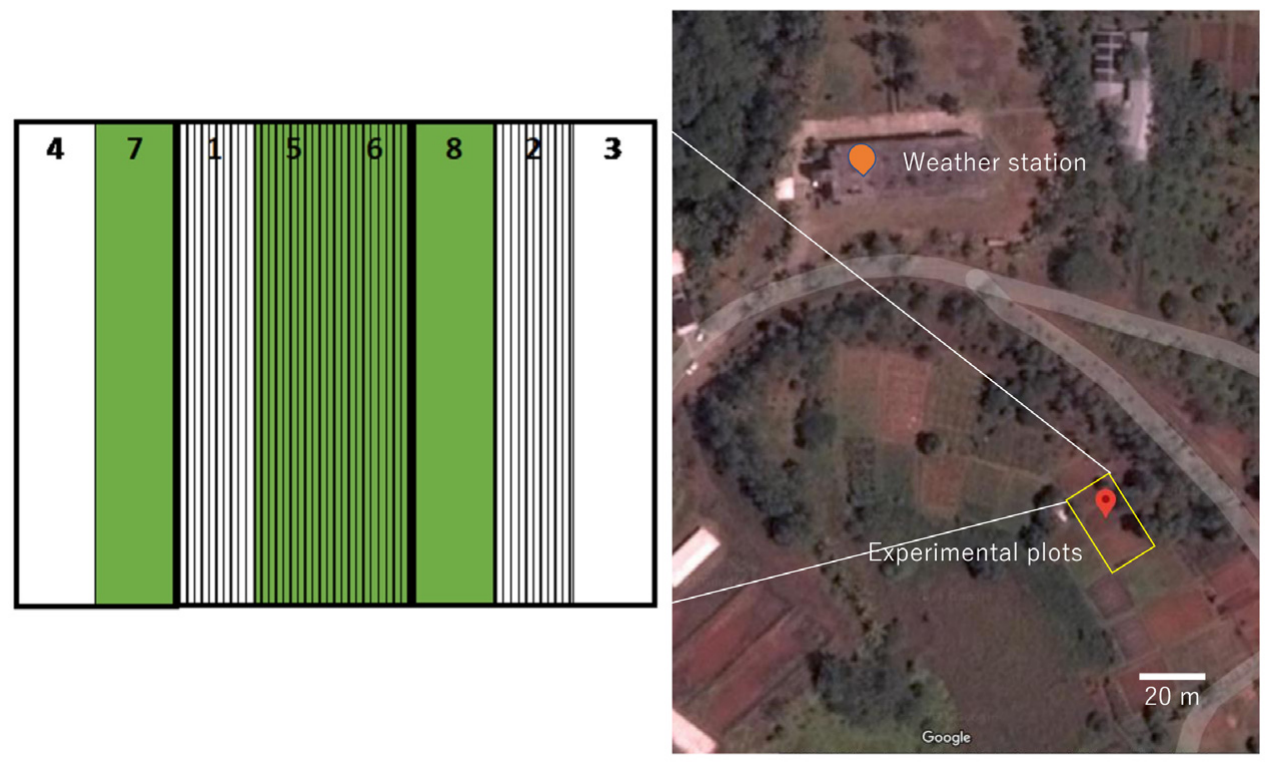
Location of plots. Green: No mulch treatment, Stripe: Ridge treatment.

**Figure 2.**
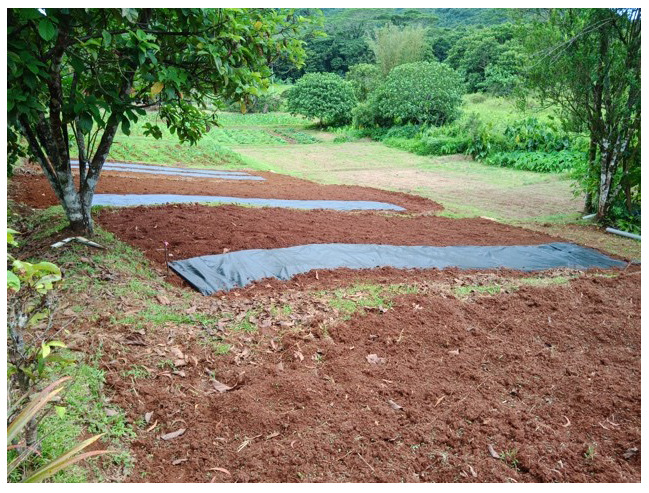
Initial condition of the field.

**Figure 3.**
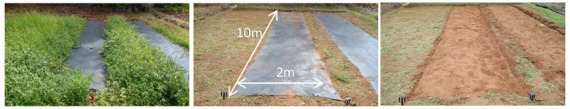
Conditions before cultivation.

**Figure 4.**
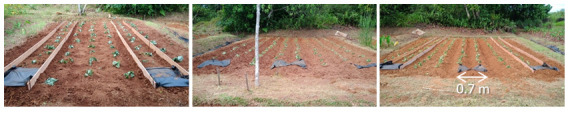
Initial conditions. The order of the plots is 4, 7, 1, 5, 6, 8, 2, 3.

We conducted the experiment from January to July 2019. The slope is 15.4° (13.4°–17.3°). The previous crop grown on the land was taro ( *Colocasia esculenta* ). The treatments were plants (with or without) × ridge (with or without) × 2 replications. We set these eight plots (2 × 10 m) randomly on the field ( Table 1, Figure 1 and Figure 2). We tilled the field using a hand tractor on January 22, leveled the field, and covered half the plots with weed control fabric (polypropylene, 0.4-mm thick, 120 g m ^–2^; I-Agri Corp., Tsuchiura) on 28 January. We cut weeds on April 16, blown off the residue, removed the weed control fabric on April 17 ( Figure 3), then tilled each plot using the hand tractor up and down to keep the soil from moving the neighboring plots. The average thickness of the soil tilled was 16 cm. We made a 70 cm width of the monitoring areas in the center of the plots by ridges or wooden boards (for the no-ridge treatment). We transplanted sweet potatoes ( *Ipomoea batatas* ) at 70 cm intervals on April 17 ( Figure 4). We dug trenches at the upper end of the fields to prevent rainwater inflow. We embanked the lower ends and added 1-m lengths of weed control fabric to trap any eroded soil. Fertilizer was not applied. Hand weeding was conducted on May 21 and June 6.

**Table 1.** Treatments.

Block	Plot ID	Plants	Ridge	Slope/°
Left	4			15.1
	7	+		17.3
Mid	1		+	14.2
	5	+	+	14.6
	6	+	+	15.7
Right	8	+		13.4
	2		+	16.1
	3			16.6

### Determination

We set up weed control fabric at the lower end of the ridge and fixed them to the ground with several wire bents into a U-shape ( Figure 4). At the bottom of the rows, we raised the soil to a height of about 20 cm to create a weir to prevent the soil from flowing out. We collected precipitation data every 5 min via a weather station in the Palau Community College Research and Development Station (about 100 m from the experimental fields; Figure 1). The condition of the fields was recorded using an automatic camera.

### Analysis

We identified rainfall events that caused severe erosion (more than 3 mm/10 min) ( Onaga, 1969) and compared the amount of eroded soil of each event.

The amount of eroded soil was predicted with the Universal Soil Loss Equation (USLE) equation ( Wischmeier & Smith, 1978) by the following formula using Microsoft Excel (Changed the original formula to meters).

A = R K LS P C metric ton ha ^–1^ year ^–1^

Where A = computed soil loss per unit area, R = the rainfall and runoff factor, K = the soil erodibility factor, LS = the topographic factor, C = the cover and management factor, and P = the support practice factor.

Storm soil losses from cultivated fields are directly proportional to a rainstorm parameter defined as the EI, and the A of each storm can be obtained using EI instead of the R.

E = 210+89 log _10_ I 100 metric ton ha ^–1^

I cm h ^–1^: maximum rainfall in 30 min multiplied to 60 min; rainfall less than 1.27 cm is omitted, and the maximum value is 7.62 cm.

Soil erosion in a rainfall event is the cumulative value.

R = EI/100 metric ton ha ^–1^

For the United States of America, for convenience, the average annual rainfall intensity for the region is prepared as a table. In the case of Palau, the frequency of rainfall is extremely high, so the integrated values during this test period are considered to be sufficiently accurate.

Finally, the above equation was parameterized for each rainfall event as follows.

A = EI K LS P C/100 metric ton ha ^–1^

K = 0.05 ( USDA Natural Resources Conservation Service; estimated based on percentage of silt, sand, and organic matter and on soil structure and permeability)

LS = (10/20.0)^0.5・(68.19 sin ^2^ 15.4° + 4.75sin 15.4°+0.068)= 4.34

C =1.0; Tillage

P = 1.00; vertical ridge

Plot area = 7 m ^2^

When the survey area is less than 10 m ^2^, the erosion rate is almost constant, but when the survey area exceeds 10 m ^2^, the erosion rate decreases linearly as the area increased ( García-Ruiz *et al.*, 2015). Accordingly, this experiment may overestimate soil erosion.

## Results

### Precipitation

**Figure 5.**
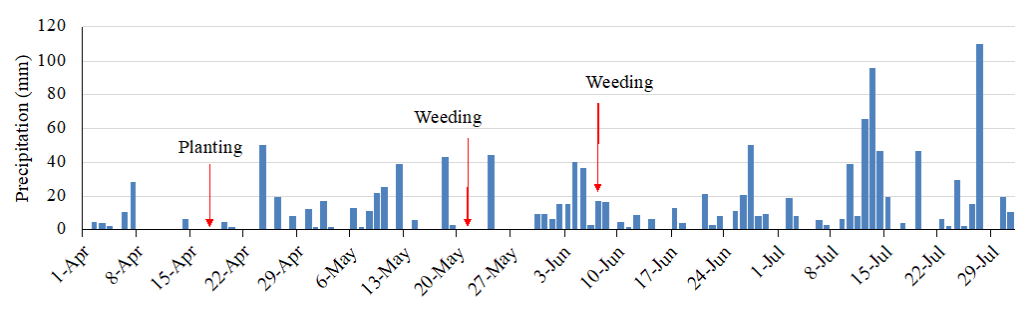
Daily precipitation.

**Figure 6.**
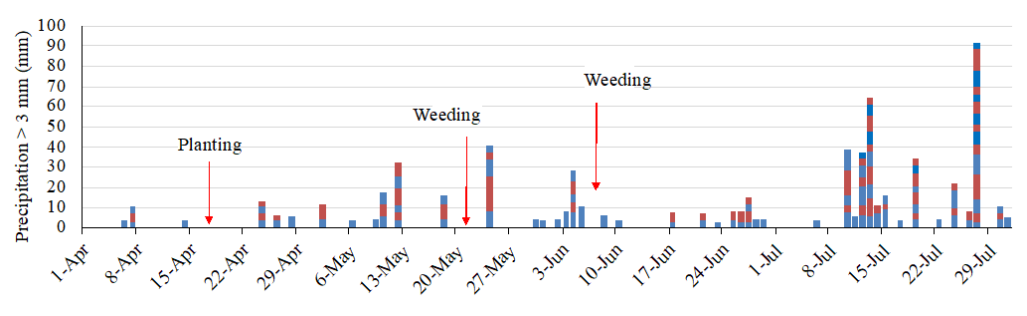
Erosive rainfall events. The blocks show a rainfall event of more than 3 mm/10 min and the amount of precipitation. The colors distinguish the events.

The field site received regular rainfall, with total precipitation of 992 mm during the experimental period, from 17 April to July 15 ( Figure 5). The field had 46 days of erosive rainfall more than 3 mm per 10 min ( Figure 6). The rainfall threshold where surface runoff occurs is 2–3 mm per 10 min on a 15° slope, although these values vary according to different soil characteristics ( Onaga, 1969). Highly erosive rainfall events occurred on day 7 after planting (May 2). The following weeding, an erosive period, a heavy rainfall event of 17 mm per 10 min occurred on the next day after weeding occurred. The second weeding was conducted after seven days of intensive rainfall, with a further erosive rainfall event of 7 mm per 10 min that occurred after weeding took place. Thus, the rainfall conditions during the experimental period were expected to result in severe soil erosion.

### Soil loss prediction by USLE

The 24 rainfall events potentially caused erosion during the observation period ( Table 2). The soil loss prediction for bare land conditions by USLE was 0.57 kg per plot on day 7 (the first rainfall event after transplanting) and 2.82 kg (after the first weeding).

**Table 2.** Soil loss prediction by USLE for each rainfall event.

Date	Day	I cm h ^–1^	E	EI	A t ha ^–1^	Erosion kg plot ^–1^	Remark
8-Apr	–9	1.76	232	4.08	0.89	0.62	(Before planting)
**24-Apr**	**7**	**1.64**	**229**	**3.76**	**0.82**	**0.57**	**1st rain**
26-Apr	9	1.36	222	3.02	0.65	0.46	
1-May	14	2.76	249	6.88	1.49	1.05	
2-May	15	2.84	250	7.11	1.54	1.08	
6-May	19	1.52	226	3.44	0.75	0.52	
9-May	22	1.68	230	3.86	0.84	0.59	
10-May	23	2.92	251	7.34	1.59	1.12	
12-May	25	3.52	259	9.10	1.98	1.38	
18-May	31	2.92	251	7.34	1.59	1.12	
**24-May**	**37**	**6.56**	**283**	**18.55**	**4.02**	**2.82**	**After weeding**
2-Jun	46	1.56	227	3.54	0.77	0.54	
3-Jun	47	2.68	248	6.65	1.44	1.01	
5-Jun	49	2.68	248	6.65	1.44	1.01	
8-Jun	52	0.76	199	1.52	0.33	0.23	After weeding
8-Jun	52	2	237	4.74	1.03	0.72	
17-Jun	61	1.72	231	3.97	0.86	0.60	
21-Jun	65	1.12	214	2.40	0.52	0.36	
27-Jun	71	1.96	236	4.63	1.00	0.70	
29-Jun	73	1.6	228	3.65	0.79	0.55	
2-Jul	76	1.04	212	2.20	0.48	0.33	
10-Jul	84	5.36	275	14.73	3.20	2.24	
13-Jul	87	2.44	244	5.97	1.29	0.91	
14-Jul	88	4.32	267	11.52	2.50	1.75	
15-Jul	89	2.44	244	5.97	1.29	0.91	(End of observation)
19-Jul	93	1.96	236	4.63	1.00	0.70	
19-Jul	93	2.36	243	5.74	1.25	0.87	
25-Jul	99	3.04	253	7.69	1.67	1.17	
26-Jul	100	2	237	4.74	1.03	0.72	
27-Jul	101	5.4	275	14.86	3.22	2.26	
28-Jul	102	4.28	266	11.39	2.47	1.73	
30-Jul	104	1.4	223	3.12	0.68	0.47	
Sum of observation period (Day 7 to 89)					32.23	22.56	

### Soil erosion

**Figure 7.**
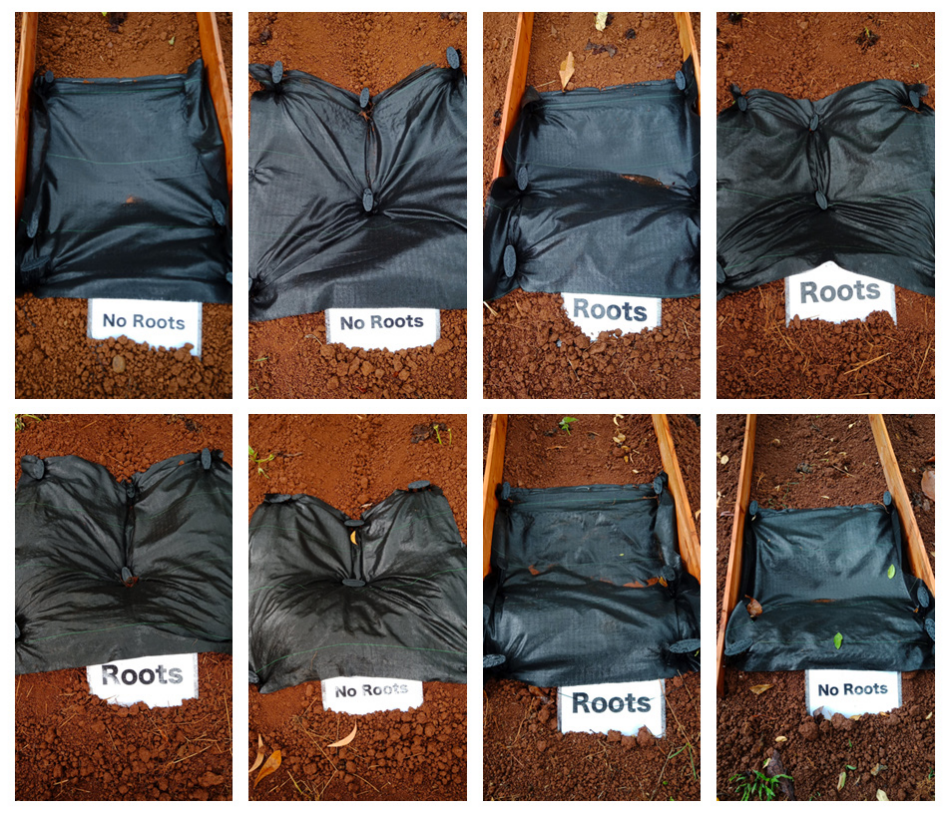
Zero erosion of the first rainfall event after transplanting. The predicted erosion was 0.57 kg per plot; however, the actual erosion was 0 kg in all plots.

Despite the severe rainfall conditions, none of the plots had any erosion at all through the experimental period ( Figure 7).

### Vegetation coverage

**Figure 8.**
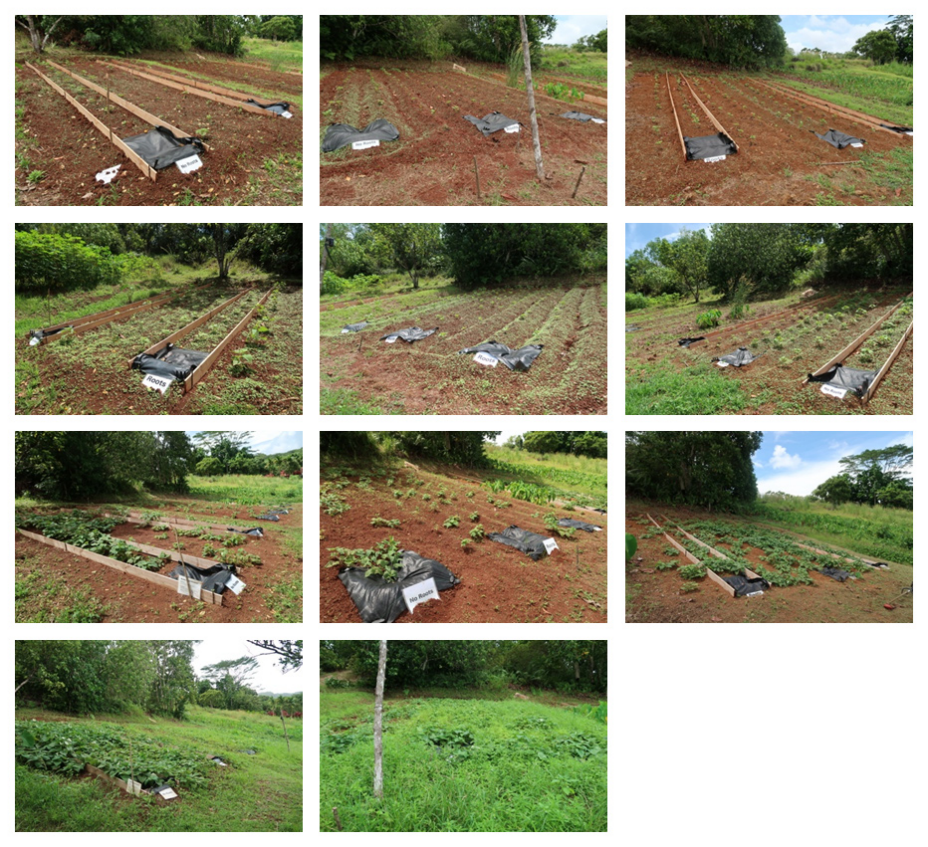
Vegetation coverage. Top panel: day 14, Upper middle panel: day 21 (before the first weeding), Lower middle panel: day 54 (after second weeding), Bottom panel day 89.

Most of the soil surface was bare by day 14 (May 1). Small weeds covered the soil surface by on day 21 (May 8), the day of the first weeding. The vegetation coverage by visual inspection ranged from 15%–85% on day 54 (Jun 10), after the second weeding. The vegetation coverage was 100% by day 89 (July15) ( Figure 8).

## Discussion and conclusion

We conducted an experiment to evaluate the effect of root mass on erosion reduction under tillage conditions. The experiment was conducted under erosion-promoting conditions: a slope of about 15°, vertical ridges, and prior placement of weed control fabric (which is expected to erase the effect of root mass in preventing soil erosion). Many intense rainfall events occurred during the experimental period, and 32 metric ton ha ^–1^ of soil erosion was predicted. Nevertheless, no soil erosion occurred. Those fields have high soil erosion tolerance and tillable. The use of mulching material expected to erase the effect of root mass of weeds for preventing soil erosion; however, our results show still the use of mulching material is available. Interestingly, soil erosion occurred in another trial conducted in an adjacent plot. They used horizontal rows. We assumed the erosion caused by catch canals. Catch canals of the horizontal ride are highly erosive ( Shima *et al.*, 1991) but vertical ridge has no catch canals.

Precipitation of 992 mm during the three months of the experimental period was large and they included 46 days of erosive rainfalls. The conditions were comparable enough to the average annual conditions of the US; therefore, the conditions were appropriate to apply the USLE equation. What is the reason for the no erosion under the condition of the large prediction and the measured eroded soil. For the parameters of the USLE equation in this study, only the K factor was not measured (cited from a USDA report). Namely, the K factor was larger than the actual value.

The estimates of K factors are based primarily on percentage of silt, sand, and organic matter and on soil structure and permeability ( USDA Natural Resources Conservation Service in 2009). Many calculation methods have been proposed for K factor such as Revised Universal Soil Loss Equation (RUSLE2), Erosion Productivity Impact Calculator (EPIC), and Geometric Mean Diameter based (Dg) model; however, the versatility of USLE for some soils are higher than that of the new method ( Wang *et al.*, 2016). Thus, there is a large room to find suitable land for agriculture from the area of specified unsuitable for agriculture. Not erosion itself, but a related story, the use of USLE to predict sediment yields is not advisable despite their present widespread application ( Boomer *et al.*, 2008).

Low erosion land for agriculture can be found by measuring erosion locally. Our results obtained from a limited field, still, this information is important for Palau's agricultural development, and the results of this test can be regarded as an example.

## Data availability

### Underlying data

Figshare: Precipitation of Palau, https://doi.org/10.6084/m9.figshare.11769909.v1 ( Oda *et al.*, 2020). Data are available under the terms of the Creative Commons Zero "No rights reserved" data waiver (CC0 1.0 Public domain dedication).
