# Supplementary material for: Locally measured USLE K factor expands sustainable agricultural land in Palau
Source: F1000Res. 2022 Jan 21;9:89. Originally published 2020 Feb 7. [Version 4] doi: 10.12688/f1000research.22229.4 (PMC8802153; doi:10.12688/f1000research.22229.4)
Supplement: Peer review history

# Peer review: Reviewer response for version 4

**DOI:** 10.5256/f1000research.119857.r120653

**Published:** 2022-01-28

**Authors:** Nor Rohaizah Jamil

**Affiliations:** 1 Department of Environment, Faculty of Forestry and Environment, Universiti Putra Malaysia, Serdang, Malaysia; 2 International Institute of Aquaculture and Aquatic Sciences (I-AQUAS), Universiti Putra Malaysia, Serdang, Malaysia

All the comments were taken into consideration in the revised version of the manuscript.

Is the work clearly and accurately presented and does it cite the current literature?

Yes

If applicable, is the statistical analysis and its interpretation appropriate?

Yes

Are all the source data underlying the results available to ensure full reproducibility?

Yes

Is the study design appropriate and is the work technically sound?

Yes

Are the conclusions drawn adequately supported by the results?

Yes

Are sufficient details of methods and analysis provided to allow replication by others?

Partly

Reviewer Expertise:

Ecohydrology, environmental flow, land use and soil erosion. I am also a certified professional for soil erosion and sediment control (CPESC).

I confirm that I have read this submission and believe that I have an appropriate level of expertise to confirm that it is of an acceptable scientific standard.

# Peer review: Reviewer response for version 3

**DOI:** 10.5256/f1000research.57917.r94091

**Published:** 2021-12-15

**Authors:** Nor Rohaizah Jamil

**Affiliations:** 1 Department of Environment, Faculty of Forestry and Environment, Universiti Putra Malaysia, Serdang, Malaysia; 2 International Institute of Aquaculture and Aquatic Sciences (I-AQUAS), Universiti Putra Malaysia, Serdang, Malaysia

1. Please remove all the personal pronouns such as "we" in the text to make it scientifically and academically-sound paper rather than of popular writing articles. Use active or passive statement (whichever is deemed more appropriate) instead.

 2. Some figures are combine-able such as Fig. 5 and 6. Suggest to consider combining them to optimize data visualisation and interpretation.

 3. Suggest to standardize the unit of precipitation in text, figures and tables.

 4. Suggested to add north arrow in the supporting figures (eg: study plot map) to enrich the information of the section.

Is the work clearly and accurately presented and does it cite the current literature?

Yes

If applicable, is the statistical analysis and its interpretation appropriate?

Yes

Are all the source data underlying the results available to ensure full reproducibility?

Yes

Is the study design appropriate and is the work technically sound?

Yes

Are the conclusions drawn adequately supported by the results?

Yes

Are sufficient details of methods and analysis provided to allow replication by others?

Partly

Reviewer Expertise:

Ecohydrology, environmental flow, land use and soil erosion. I am also a certified professional for soil erosion and sediment control (CPESC).

I confirm that I have read this submission and believe that I have an appropriate level of expertise to confirm that it is of an acceptable scientific standard, however I have significant reservations, as outlined above.

# Response

**Published:** 2021-12-28

**Authors:** Masato Oda

**Affiliations:** Japan International Research Center for Agricultural Sciences (JIRCAS), Japan

We would like to express our sincere gratitude for your review of our manuscript.

**1. Removing all personal pronouns.**

- All the Methods and the Result expressions were fixed. However, some sentences in the other sections have not been changed because they were considered appropriate (for example, for avoiding long subjects).

**2. Combine-able figures.**

- A part of illustration in Figure 1 was replaced with Figures.
- The illustration combined with Figures 4.
- Figure 5 was combined with Figure 6.

**3. Standardize the unit of precipitation.**

- The “cm” in the Methods and Analysis are citations, so we left them as they are. Using "mm" has a problem of accuracy. We added a footnote to Table 2.

**4. Adding north arrow in the supporting figures (eg: study plot map).**

- We added that to Figure 1.

**5. Additional revise**

- We found mistakes in the number of erosive rainfall events in the results section and the discussion section, and corrected them from 46 to 24. 
- In addition, minor grammar corrections have been made.

# Peer review: Reviewer response for version 2

**DOI:** 10.5256/f1000research.27523.r83162

**Published:** 2021-05-06

**Authors:** Kebede Wolka

**Affiliations:** 1 Wondo Genet College of Forestry and Natural Resource, Hawassa University, Shashemene, Ethiopia

The research attempted to show the K-factor of the universal soil equation in Palau. In the study design, a small plot of about 20m ^2^ used to collect the eroded material, which the study reported no erosion despite a reasonable amount of rainfall. The study is conducted in areas where crop growth is less common. The research approach is good to show erosion under specific conditions. Like many other researches, it has shortcomings, specified below:

1. Method of calculation for USLE has not been mentioned clearly.
2. The study reported that the K-factor overestimated the soil loss in USLE. However, there are options to consider K-factor for different soil. The options followed to use K-factor has not been mentioned, which might be inappropriate that led to far overestimating result.
3. The plot considered in the experiment seems smaller.
4. Results of the study has not been discussed sufficiently and fully.
5. In most cases, the USLE is meant to estimate soil loss at annual base. The author estimated the soil loss for every rainfall event. How reliable is this method?
6. The paper has a few editing problems.

Is the work clearly and accurately presented and does it cite the current literature?

Partly

If applicable, is the statistical analysis and its interpretation appropriate?

Not applicable

Are all the source data underlying the results available to ensure full reproducibility?

Partly

Is the study design appropriate and is the work technically sound?

Partly

Are the conclusions drawn adequately supported by the results?

Yes

Are sufficient details of methods and analysis provided to allow replication by others?

No

Reviewer Expertise:

Erosion and soil and water conservation

I confirm that I have read this submission and believe that I have an appropriate level of expertise to confirm that it is of an acceptable scientific standard, however I have significant reservations, as outlined above.

# Response

**Published:** 2021-06-17

**Authors:** Masato Oda

**Affiliations:** Japan International Research Center for Agricultural Sciences (JIRCAS), Japan

Thank you for reviewing our manuscript. We have dealt with the matters you pointed out as follows. Please check the revised manuscript.

1. *Method of calculation for USLE has not been mentioned clearly.*
**A) We made the method citation more faithful and detailed. We also clarified that the rainfall intensity was calculated according to the definition. Relatedly, we corrected the symbols in the formula.**
2. *The study reported that the K-factor overestimated the soil loss in USLE. However, there are options to consider the K-factor for different soil. The options followed to use K-factor have not been mentioned, which might be inappropriate that led to far overestimating the result.*
**A) In the abstract, methods, and discussion sections, we referred to that the K-factor was estimated based on soil texture and permeability.**
**  **
3. *The plot considered in the experiment seems smaller.*
**A) When the survey area is less than 10 m2, the erosion rate is almost constant, but when the survey area exceeds 10 m2, the erosion rate decreases linearly as the area increased. Accordingly, this experiment may overestimate soil erosion. We quoted the literature in the method section.**
**  **
4. *Results of the study has not been discussed sufficiently and fully.*
**A) The flow of discussion has been improved as follows. 1) Summarizing the results of the experiment, 2) Considering that the experiment meets the conditions for applying the USLE prediction formula, 3) Pointing out the gap between the predicted value and the measured value, and that the gap is derived from K-factor, 4) Showing that various improvement proposals have been shown for K factor, but there are cases where the original is more accurate depending on the soil, and the definitive calculation method has not been determined. 5 ) As a result of the above, there is a good possibility of finding suitable land in an unsuitable land for agriculture in Palau, and the results of this test can be regarded as an example.**
5. *In most cases, the USLE is meant to estimate soil loss at annual base. The author estimated the soil loss for every rainfall event. How reliable is this method?*
**A) Clarified in the method section that the calculation is based on the USLE forecast formula. We also mentioned in the discussion that the number of events is considered to be large enough.**

# Peer review: Reviewer response for version 1

**DOI:** 10.5256/f1000research.24517.r61753

**Published:** 2020-04-30

**Authors:** Kathleen B. Boomer

**Affiliations:** 1 Foundation for Food and Agriculture Research, Washington, DC, USA; 2 CBP Scientific Technical Advisory Committee, Washington, DC, USA

Technical Review: Use of the K factor from the Universal Soil Loss Equation can show arable land in Palau by Oda *et al.*

**General Comments:**

 The investigators report the results of measured and estimated soil erosion in the Micronesia region of the western Pacific Ocean. To compare how plantings and micro-tilling affects soil detachment and transport to field edges, they established eight plots within a single field, following as closely as possible (presumably) the original protocol of the USLE. Although the title indicates a focus on soil erodibility (K), the study design is more relevant to understanding practice effects on erosion. Given the importance of subsistence agriculture and the lack of studies specific to promoting sustainable practices in this region, this work calls attention to a critical information gap.

 The manuscript requires extensive revisions to clarify and substantiate its contributions. It lacks a “story arc” that enables the reader to readily understand why this work is important, how the researchers tackled this knowledge gap, and how their results compare or contribute to the existing body of research. For example, the authors might consider highlighting the importance of subsistence farming to the region and the challenge of soil erosion to sustainable agriculture, but the lack of technical guidance specific to the region. The USLE, which is based on a phenomenal US dataset, is a widely accepted tool to evaluate erosion potential based on landscape setting and management, but the empirical model ideally requires local testing and refinements for application to areas outside of its original US study area. The research objectives and methods also require clarification. Finally, the limited number of plots (8) and fields (1) raises concerns about the power of the study to detect differences among field practices. Given the difficulty and expense of field studies and the realities of scant data, however, the publication may be valuable.

**Specific Comments:**

- A large portion of the introduction includes study site information, which should go in the Methods section.
- Use the international the international system for classification of soils to describe site conditions.
- Define “T factor”.
- In the site description, add information about the landscape setting, outside of the study site boundaries. A study site topographic map would be helpful. Information about the distribution of croplands on the study island, as well as a brief description of how the island compares to others in the region, would be helpful to thinking about the value of information beyond the study site.
- In the methods section, add a paragraph describing how and where rainfall data were collected. Include this location on the study area map.
- It is not clear how tilling, even if by hand, does not mix soils.
- Elaborate on how soil erosion was measured. Reference methods to the extent possible.
- Elaborate on USLE application and parameterization. Use previous publications as examples.
- In the “Estimated erosion” section, define “That” in the last sentence on page 3.
- Consolidate Figure 6 into one panel.
- In the Discussion and Conclusions section, paragraph 1, reference to site conditions seem contradictory: “experiment was under sever conditions, with a slope of … 15°” vs “risk of soil erosion was low for the experimental soil”.
- Convert Table 2 to graphical form. For example, plot A vs EI, observed vs predicted, grouped by practice.
- The pictures are difficult to interpret.
- The discussion needs to follow the same “story arc” presented in the introduction and used to organize the methods and results sections.The discussion also needs to compare the study results with the existing body of literature to assess whether findings to reinforce or challenge existing paradigms.The authors also need to acknowledge the limitations of their study design and ideally, discuss the relevance of their findings to management recommendations.

Is the work clearly and accurately presented and does it cite the current literature?

No

If applicable, is the statistical analysis and its interpretation appropriate?

No

Are all the source data underlying the results available to ensure full reproducibility?

Partly

Is the study design appropriate and is the work technically sound?

Partly

Are the conclusions drawn adequately supported by the results?

No

Are sufficient details of methods and analysis provided to allow replication by others?

No

Reviewer Expertise:

Hydrology, soil biogeochemistry, and watershed modeling

I confirm that I have read this submission and believe that I have an appropriate level of expertise to confirm that it is of an acceptable scientific standard, however I have significant reservations, as outlined above.

# Response

**Published:** 2020-05-25

**Authors:** Masato Oda

**Affiliations:** Japan International Research Center for Agricultural Sciences (JIRCAS), Japan

Thank you for the constructive comments.

We will accept almost of all the comments; however, before submitting a revised manuscript, may I clarify the following points?

**1) Consolidate Figure 6 into one panel.**

Is that mean combine Figure 6 and Figure 5?

**2) Convert Table 2 to graphical form. For example, plot A vs EI, observed vs predicted, grouped by practice.** 

This is less effective because the practical data were all zero.

# Response

**Published:** 2020-06-02

**Authors:** Masato Oda

**Affiliations:** Japan International Research Center for Agricultural Sciences (JIRCAS), Japan

**Define “T factor”.**

 Now, I understood that I misunderstood the meaning of the "T factor".

 Thank you so much.

# Response

**Published:** 2020-06-19

**Authors:** Masato Oda

**Affiliations:** Japan International Research Center for Agricultural Sciences (JIRCAS), Japan

Clarified the “story arc” of the paper including the lack of technical guidance specific to the region, the research objectives, and methods.

1. The description of Palau was moved to the Methods section.
2. The international classification of soils was added.
3. Define of the “T factor” was corrected.
4. A Google map link was added as an alternative of the topographic map
5. The information on the experimental field was added.
6. The distance of the weather station was added.
7. The link of a picture of the hand tractor is added.
8. The method of collecting eroded soil was clarified more.
9. The application and parameterization of USLE were clarified more.
10. “That” in the last sentence on page 3 was clarified.
11. We couldn’t understand the meaning of “Consolidate Figure 6 into one panel”.
12. We improved the readability. The first paragraph pointed out a contradiction and interpreted that in the second and the third paragraph.
13. No erosion was observed so the changing Table 2 to graphical form is ineffective.
14. We corrected estimated → predicted, actual was zero.
15. The discussion section was improved.
